# Disruption of Apicoplast Biogenesis by Chemical Stabilization of an Imported Protein Evades the Delayed-Death Phenotype in Malaria Parasites

**DOI:** 10.1128/mSphere.00710-18

**Published:** 2019-01-23

**Authors:** Michael J. Boucher, Ellen Yeh

**Affiliations:** a Department of Microbiology and Immunology, Stanford University School of Medicine, Stanford, California, USA; b Department of Biochemistry, Stanford University School of Medicine, Stanford, California, USA; c Department of Pathology, Stanford University School of Medicine, Stanford, California, USA; d Chan Zuckerberg Biohub, San Francisco, California, USA; University at Buffalo

**Keywords:** *Plasmodium*, apicoplast, malaria, organelle protein import

## Abstract

Malaria parasites (*Plasmodium* spp.) contain a nonphotosynthetic plastid organelle called the apicoplast, which houses essential metabolic pathways and is required throughout the parasite life cycle. The biogenesis pathways responsible for apicoplast growth, division, and inheritance are of key interest as potential drug targets. Unfortunately, several known apicoplast biogenesis inhibitors are of limited clinical utility because they cause a peculiar “delayed-death” phenotype in which parasites do not stop replicating until the second lytic cycle posttreatment. Identifying apicoplast biogenesis pathways that avoid the delayed-death phenomenon is a priority. Here, we generated parasites targeting a murine dihydrofolate reductase (mDHFR) domain, which can be conditionally stabilized with the compound WR99210, to the apicoplast. Surprisingly, chemical stabilization of this exogenous fusion protein disrupted parasite growth in an apicoplast-specific manner after a single lytic cycle. WR99210-treated parasites exhibited an apicoplast biogenesis defect beginning within the same lytic cycle as drug treatment, indicating that stabilized mDHFR perturbs a non-delayed-death biogenesis pathway. While the precise mechanism-of-action of the stabilized fusion is still unclear, we hypothesize that it inhibits apicoplast protein import by stalling within and blocking translocons in the apicoplast membranes.

**IMPORTANCE** Malaria is a major cause of global childhood mortality. To sustain progress in disease control made in the last decade, new antimalarial therapies are needed to combat emerging drug resistance. Malaria parasites contain a relict chloroplast called the apicoplast, which harbors new targets for drug discovery. Unfortunately, some drugs targeting apicoplast pathways exhibit a delayed-death phenotype, which results in a slow onset-of-action that precludes their use as fast-acting, frontline therapies. Identification of druggable apicoplast biogenesis factors that will avoid the delayed-death phenotype is an important priority. Here, we find that chemical stabilization of an apicoplast-targeted mDHFR domain disrupts apicoplast biogenesis and inhibits parasite growth after a single lytic cycle, suggesting a non-delayed-death target. Our finding indicates that further interrogation of the mechanism-of-action of this exogenous fusion protein may reveal novel therapeutic avenues.

## Abstract (precis)

Malaria is a major cause of global childhood mortality. To sustain progress in disease control made in the last decade, new antimalarial therapies are needed to combat emerging drug resistance. Malaria parasites contain a relict chloroplast called the apicoplast, which harbors new targets for drug discovery. Unfortunately, some drugs targeting apicoplast pathways exhibit a delayed-death phenotype, which results in a slow onset-of-action that precludes their use as fast-acting, frontline therapies. Identification of druggable apicoplast biogenesis factors that will avoid the delayed-death phenotype is an important priority. Here, we find that chemical stabilization of an apicoplast-targeted mDHFR domain disrupts apicoplast biogenesis and inhibits parasite growth after a single lytic cycle, suggesting a non-delayed-death target. Our finding indicates that further interrogation of the mechanism-of-action of this exogenous fusion protein may reveal novel therapeutic avenues.

## INTRODUCTION

*Plasmodium* parasites cause malaria and are responsible for over 200 million human infections and over 400,000 deaths annually (1). Despite a reduction in malaria-related mortality in the past 15 years, emerging resistance to frontline antimalarials necessitates continued development of new chemotherapies (2, 3). One key source of drug targets is the apicoplast, a nonphotosynthetic plastid organelle found in many apicomplexan pathogens (4, 5). The apicoplast produces essential metabolites required for parasite survival throughout its life cycle (6). Derived from secondary endosymbiosis of an ancestral red alga, the apicoplast is surrounded by 4 membranes and utilizes a complex but poorly understood set of biogenesis pathways to carry out organelle growth, division, and inheritance (7). These pathways are of particular interest as drug targets due to their importance for parasite replication and distinction from human host pathways. Indeed, apicoplast DNA replication and protein translation are validated targets of small-molecule inhibitors (8–12).

Confirming the utility of apicoplast biogenesis as a drug target, the translation inhibitors doxycycline and clindamycin are in clinical use as a prophylactic and partner drug, respectively (13–15). However, one important limitation of these and other apicoplast “housekeeping” inhibitors is that they result in a peculiar “delayed-death” phenotype (9, 10). During delayed death, parasite growth is unaffected after the first lytic cycle of inhibitor treatment but is inhibited following the second lytic cycle, even after drug removal. This *in vitro* phenotype manifests as a slow onset-of-action that limits clinical use of these drugs. While inhibitors that act on a faster timescale are clearly desirable, only 1 apicoplast biogenesis inhibitor, actinonin, is known to avoid the delayed-death phenotype in malaria parasites (12, 16, 17). Furthermore, our poor mechanistic understanding of delayed death makes it difficult to assess *a priori* which biogenesis pathways might display this phenotype. While conditional genetic tools could provide an avenue to test potential targets for delayed death, most tools for *Plasmodium* parasites act at the DNA or RNA levels (18) and do not necessarily recapitulate growth inhibition kinetics of direct chemical inhibition of that same target (17, 19, 20). Destabilization domains that conditionally target proteins for degradation by the cytosolic ubiquitin-proteasome enable protein-level disruption (21, 22), but these systems are not suitable to study apicoplast-localized proteins, which are inaccessible to the cytosolic proteasome.

A murine dihydrofolate reductase (mDHFR) domain that can be conditionally stabilized by a small molecule has been used to characterize protein translocation steps during yeast mitochondrial protein import *in vitro* (23) and, more recently, during export of *Plasmodium* proteins across the parasitophorous vacuole (PV) membrane into the host red blood cell (24–26). Here, while generating a Plasmodium falciparum cell line expressing an apicoplast-targeted mDHFR domain for other purposes, we unexpectedly observed that stabilization with the compound WR99210 caused parasite death after a single lytic cycle. While the precise mechanism-of-action is unclear, chemical stabilization induces an apicoplast biogenesis defect that emerges within the same replication cycle, suggesting that it disrupts an important apicoplast biogenesis pathway. These results indicate that further study of the mechanism for this biogenesis defect may identify apicoplast pathways that avoid the delayed-death phenotype.

## RESULTS

**FIG 1:**
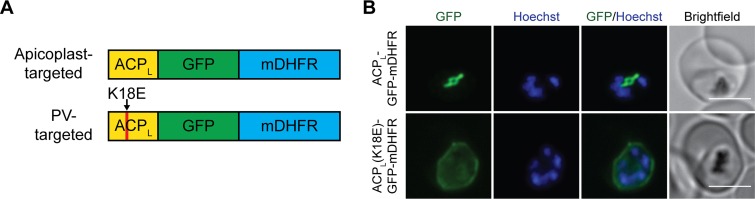
Generation of parasites expressing GFP-mDHFR fusions in the apicoplast or PV. (A) Schematic (not to scale) of constructs targeting GFP-mDHFR to the apicoplast via the ACP leader sequence (first 55 amino acids of ACP) or to the PV via a mutant (K18E) ACP leader sequence. (B) Live-cell imaging of GFP-mDHFR fusions. Nuclei were stained with Hoechst 33342. Brightness/contrast adjustments were not held constant between the two cell lines due to differences in GFP fluorescence intensity in the apicoplast versus the PV. Bars, 5 µm.

We generated parasites that target a GFP-mDHFR fusion to the apicoplast using the apicoplast-targeting leader sequence (first 55 amino acids) of acyl carrier protein (ACP) (Fig. 1A) (27, 28). We also generated negative-control parasites expressing a version of this fusion in which lysine 18 of the ACP leader sequence was mutated to glutamate (K18E). This mutation renders the apicoplast targeting sequence nonfunctional and causes mistargeting of proteins to the PV (29). Both fusion proteins were expressed in P. falciparum Dd2^attB^ parasites (30) and localized to the expected compartments (Fig. 1B).

**FIG 2:**
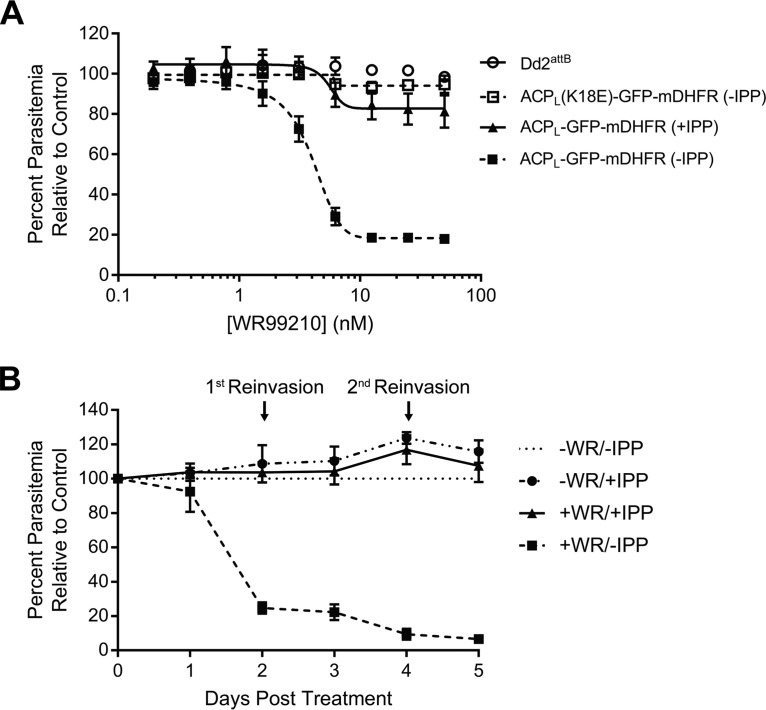
Stabilization of ACP_L_-GFP-mDHFR causes apicoplast-specific growth inhibition after the first lytic cycle. (A) Growth of parental Dd2^attB^, ACP_L_-GFP-mDHFR, and ACP_L_(K18E)-GFP-mDHFR parasites after 3 days in response to increasing doses of WR99210. ACP_L_-GFP-mDHFR parasites were assayed in both the presence and absence of 200 µM IPP. (B) Growth of ACP_L_-GFP-mDHFR parasites in the presence of 10 nM WR99210, 200 μM IPP, or both over a 5-day time course. Parasitemia was normalized to the -WR/-IPP control at each time point. Error bars in both panels represent standard deviation of the mean from 3 biological replicates. Biological replicates in panel A were performed in technical triplicate.

Surprisingly, we found that addition of WR99210 to parasites expressing ACP_L_-GFP-mDHFR resulted in dose-dependent growth inhibition in a 3-day (1-lytic-cycle) growth assay (Fig. 2A, closed squares). We confirmed that parental Dd2^attB^ parasites, which are resistant to WR99210 due to expression of a human DHFR allele, were unaffected at the WR99210 concentrations tested (Fig. 2A, open circles). Furthermore, we found that parasites expressing the PV-targeted ACP_L_(K18E)-GFP-mDHFR construct remained insensitive to WR99210 (Fig. 2A, open squares), indicating that growth inhibition was specifically due to apicoplast targeting and not general toxicity of the stabilized fusion protein.

During *in vitro* culture of blood-stage P. falciparum, the isoprenoid precursor isopentenyl pyrophosphate (IPP) is the only essential metabolic product of the apicoplast. As such, supplementation with exogenous IPP can rescue apicoplast defects and can be used to identify apicoplast-specific phenotypes (11). IPP supplementation reversed the WR99210 sensitivity of ACP_L_-GFP-mDHFR parasites both in a 3-day dose-response assay and over a 5-day time course (Fig. 2A and B), confirming that the WR99210 sensitivity conferred by ACP_L_-GFP-mDHFR is specifically due to disruption of an apicoplast pathway.

**FIG 3:**
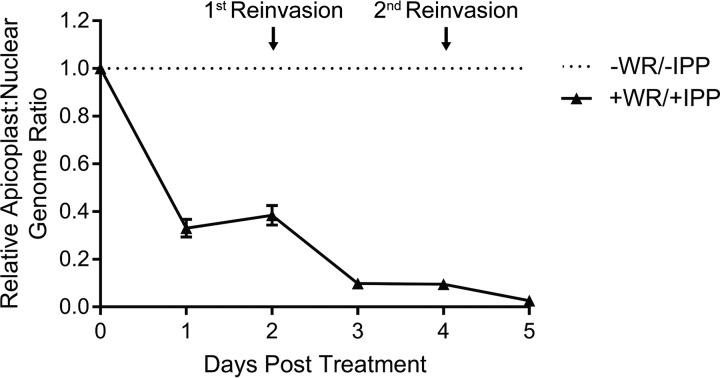
Relative abundance of the apicoplast genome following ACP_L_-GFP-mDHFR stabilization. The relative apicoplast/nuclear genome ratio of ACP_L_-GFP-mDHFR parasites grown in 10 nM WR99210 and 200 µM IPP was measured by qPCR over 5 days of treatment. Values are normalized to the −WR/−IPP control at each time point. Error bars represent standard deviation of the mean from 3 biological replicates, each analyzed in technical triplicate.

Two categories of IPP-rescuable apicoplast defects have been characterized: (i) inhibition of apicoplast biogenesis, during which apicoplast growth, division, and inheritance are disrupted and lead to organelle loss, and (ii) inhibition of isoprenoid biosynthesis, during which the metabolic function of the apicoplast is blocked but the organelle itself remains intact (11, 12, 17, 31, 32). To distinguish these possibilities, we performed 3 assays to assess the status of the apicoplast in WR99210-treated, ACP_L_-GFP-mDHFR parasites. First, we used quantitative PCR (qPCR) to measure the relative abundance of the apicoplast genome, which is lost when apicoplast biogenesis is perturbed (11). We found that WR99210-treated, IPP-rescued parasites exhibited a decrease in their apicoplast/nuclear genome ratio after 1 day of WR99210 treatment with further decreases after 3 and 5 days of treatment (Fig. 3). Because the apicoplast/nuclear genome ratio is a relative measurement, the drop in this value observed after 1 day of WR99210 treatment could be due to either (i) a decrease in the levels of the apicoplast genome (i.e., degradation) or (ii) a lack of apicoplast DNA replication concomitant with the increase in nuclear DNA copy number that occurs in trophozoite- and schizont-stage parasites. This result suggests a biogenesis defect that emerges as early as 1 day after ACP_L_-GFP-mDHFR stabilization.

**FIG 4:**
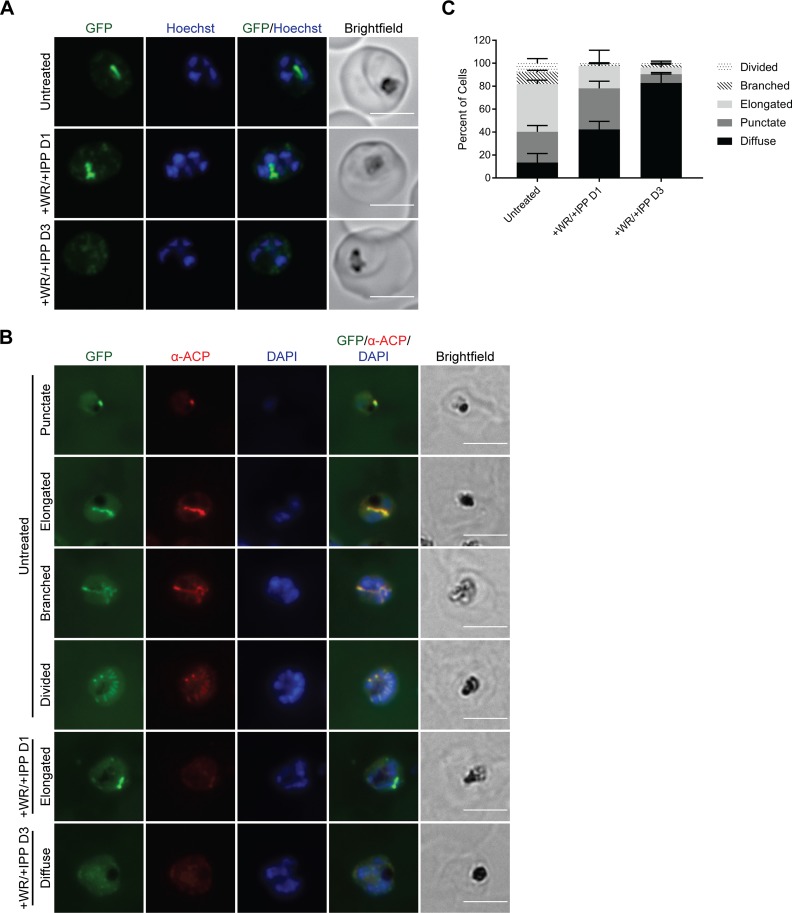
Stabilization of ACP_L_-GFP-mDHFR disrupts localization of endogenous apicoplast cargo. ACP_L_-GFP-mDHFR parasites were grown with 10 nM WR99210 and 200 µM IPP for either 1 or 3 days. (A) Live imaging of Hoechst 33342-stained parasites. Bars, 5 µm. (B) Fixed imaging of parasites stained with an antibody against the endogenous apicoplast marker ACP. The ACP antibody was raised against amino acids 57 to 137 of ACP (58) and therefore recognizes epitopes distinct from the first 55 amino acids that constitute the ACP leader sequence. Bars, 5 µm. (C) Quantification of localization patterns in panel B. Error bars represent standard deviation of the mean from 2 biological replicates. A minimum of 100 cells were scored for each condition of each replicate.

Next, we characterized the localization of the ACP_L_-GFP-mDHFR fusion protein and the endogenous apicoplast protein ACP following WR99210 treatment. One day posttreatment, live imaging confirmed that WR99210-stabilized ACP_L_-GFP-mDHFR displayed an apicoplast-like localization pattern (Fig. 4A). Three days posttreatment, ACP_L_-GFP-mDHFR was observed in a pattern of diffuse puncta resembling that of mislocalized nucleus-encoded apicoplast proteins when parasites have lost their apicoplast (Fig. 4A) (11, 31). Importantly, these results indicate that WR99210 stabilization did not result in unexpected localization of the fusion protein.

To assess how ACP_L_-GFP-mDHFR stabilization affected endogenous cargo, we performed immunofluorescence analysis (IFA) to characterize the localization of endogenous ACP after 1 or 3 days of WR99210 treatment. We manually classified ACP localization in individual cells as either the previously described “punctate,” “elongated,” “branched,” or “divided” patterns of intact plastids (33) or the “diffuse” pattern observed in parasites lacking their plastids (11) (Fig. 4B and C). As expected, most untreated parasites exhibited ACP localization patterns consistent with the established apicoplast developmental stages (Fig. 4C). Some untreated parasites displayed a diffuse localization pattern that suggests loss of the plastid, and we expect that this is due to either (i) poor penetration of the ACP antibody into these cells, manifesting similarly to the diffuse staining of plastidless parasites, or (ii) bona fide apicoplast loss due to WR99210-independent mDHFR stabilization, which was observed in previous studies targeting mDHFR for export from Toxoplasma gondii (34, 35). Regardless, we noted an increase in the number of parasites with diffuse ACP staining after 1 and 3 days of WR99210 treatment (Fig. 4C), indicating a biogenesis defect at these time points. Additionally, we observed that even some parasites with intact plastids after 1 day of WR99210 treatment exhibited decreased ACP staining in the apicoplast, potentially indicating an intermediate phenotype at this earlier time point (Fig. 4B, +WR/+IPP D1 panel).

**FIG 5:**
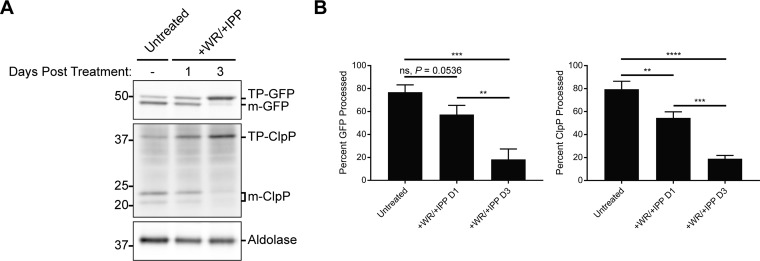
Stabilization of ACP_L_-GFP-mDHFR disrupts transit peptide processing of endogenous apicoplast cargo. ACP_L_-GFP-mDHFR parasites were grown with 10 nM WR99210 and 200 µM IPP for either 1 or 3 days. (A) Western blotting to assess transit peptide processing of ACP_L_-GFP-mDHFR and the endogenous apicoplast protein ClpP. TP, transit peptide; m-, mature (processed) protein. Numbers at left, molecular masses in kDa. (B) Quantification of transit peptide processing for ACP_L_-GFP-mDHFR and ClpP. Data are expressed as the percentage of total GFP or ClpP signal that is mature (processed). Error bars represent standard deviation of the mean from 3 biological replicates. **, *P < *0.01; ***, *P < *0.001; ****, *P < *0.0001, one-way ANOVA with Tukey’s multiple-comparison test. ns, not significant.

Finally, most apicoplast-targeted proteins contain an N-terminal transit peptide that is proteolytically processed upon successful import, and an accumulation of unprocessed protein can be used as a marker for the loss of protein import that accompanies apicoplast loss (11). We therefore assessed the processing of the ACP_L_-GFP-mDHFR fusion and the endogenous apicoplast protein ClpP by Western blotting. Consistent with a defect in protein import, WR99210-treated parasites exhibited a modest but reproducible accumulation of unprocessed ACP_L_-GFP-mDHFR after 1 day of WR99210 treatment (Fig. 5A and B). ClpP showed a comparable transit peptide processing defect (Fig. 5A and B), confirming that stabilization of ACP_L_-GFP-mDHFR affected import not only of the fusion protein itself but also of endogenous cargo. The unprocessed ClpP isoform we observed migrated similarly to unprocessed ClpP in parasites whose plastids were ablated by a 3-day treatment with actinonin (see Fig. S1 in the supplemental material), confirming that the unprocessed ClpP observed is not a previously reported isoform that lacks the transit peptide but retains a prodomain (36). Processing of both ACP_L_-GFP-mDHFR and ClpP was almost completely ablated after 3 days of WR99210 treatment (Fig. 5A and B). Altogether, WR99210-treated ACP_L_-GFP-mDHFR parasites display a loss in their apicoplast/nuclear genome ratios, mislocalization of endogenous cargo, and a defect in transit peptide processing, confirming that ACP_L_-GFP-mDHFR stabilization results in an apicoplast biogenesis defect.

10.1128/mSphere.00710-18.1 FIG S1 Comparison of ClpP processing after ACP_L_-GFP-mDHFR stabilization or actinonin treatment. ACP_L_-GFP-mDHFR parasites were grown with 10 nM WR99210 and 200 µM IPP for either 1 or 3 days or with 10 µM actinonin and 200 µM IPP for 3 days. Lysates were blotted for ClpP. TP, transit peptide; m-, mature (processed) protein. Download FIG S1, TIF file, 0.5 MB. Copyright © 2019 Boucher and Yeh. 2019 Boucher and Yeh This content is distributed under the terms of the Creative Commons Attribution 4.0 International license.

## DISCUSSION

Here, we show that stabilization of an exogenous GFP-mDHFR fusion targeted to the P. falciparum apicoplast disrupts parasite growth after a single lytic cycle (Fig. 2) and that this growth defect is specifically due to a block in apicoplast biogenesis (Fig. 3, 4, and 5). Apicoplast biogenesis defects begin after 1 day of WR99210 treatment and are more pronounced after 3 days of treatment. The fact that all 3 phenotypes assayed emerge at an early time point suggests that ACP_L_-GFP-mDHFR stabilization affects a central process, ablation of which rapidly affects apicoplast biogenesis and function.

Two models may explain why ACP_L_-GFP-mDHFR stabilization disrupts apicoplast biogenesis. First, similar to a previous study showing that stabilization of an SBP1-mDHFR-GFP fusion blocks translocation of P. falciparum proteins to the red blood cell cytosol and halts parasite growth (26), stabilization of ACP_L_-GFP-mDHFR might prevent import of apicoplast cargo by blocking putative apicoplast translocons. Indeed, import of more than 300 nucleus-encoded apicoplast proteins is thought to involve homologs of the translocation and ubiquitylation machinery typically involved in ER-associated degradation (ERAD) (37–41) as well as homologs of the translocons on the outer and inner chloroplast membranes (TOC and TIC complexes) of primary plastids (38, 42–44). Given the context of previous uses of mDHFR to block protein translocation in apicomplexan parasites (24–26, 34, 35), stalling of mDHFR in apicoplast translocons is a parsimonious model explaining the data presented here. Potentially inconsistent with this model, however, a significant amount of both ACP_L_-GFP-mDHFR and ClpP still undergoes transit peptide processing after 1 day of WR99210 treatment (Fig. 5). Nonetheless, we expect that this may simply indicate that (i) some processed protein was already present in the apicoplast prior to WR99210 treatment or (ii) mDHFR stabilization does not result in a complete, irreversible block in protein import but rather delays the kinetics of protein import. Regardless, because we could not detect biochemical interaction of ACP_L_-GFP-mDHFR with putative apicoplast translocons, we cannot definitively conclude that a protein import defect is the reason that stabilized ACP_L_-GFP-mDHFR disrupts apicoplast biogenesis.

Given the lack of direct evidence for a protein import defect, an alternative and non-mutually exclusive model is that stabilization of ACP_L_-GFP-mDHFR causes general apicoplast toxicity. While the nonspecific nature of this model makes it difficult to test directly, it is reasonable to hypothesize that the presence of a strongly folded, nonnative protein domain in the organelle lumen is sufficient to disrupt one or more organelle biogenesis pathways. This might occur, for example, by aberrant interaction with key biogenesis factors in a manner that inhibits their normal functions or by overtaxing the ClpCP protease system that is thought to mediate protein quality control in the apicoplast.

Regardless of the mechanism by which ACP_L_-GFP-mDHFR causes apicoplast loss, our findings have important implications for identifying and prioritizing antimalarial drug targets. The delayed-death phenotype is a key limitation of known apicoplast biogenesis inhibitors, and future work should focus on targets that will avoid this phenomenon. While many conditional genetic tools do not act rapidly enough to reveal such targets, our use of a conditionally stabilizable mDHFR domain highlights the utility of protein-level tools. Future efforts to develop such tools for generalized use in the apicoplast may enable perturbations that will more precisely mimic direct chemical inhibition. While current destabilization domains applied to P. falciparum use conditionally unfoldable FKBP or DHFR domains to target proteins to the cytosolic proteasome (21, 22), one could imagine engineering a conditional degradation system based on a degron that functions in the apicoplast, such as the Escherichia coli SsrA degron (45).

Second, the models described above suggest 2 biogenesis pathways that may avoid the delayed-death phenotype: the Clp protease system and apicoplast protein import. Given the potential of bacterial Clp components as drug targets (46) and the importance of the Clp protease system for apicoplast biogenesis (36), this pathway could be a valuable target if it does indeed avoid the delayed-death phenotype. Apicoplast protein import presents an even larger array of potential targets, as over a dozen proteins have been implicated in this pathway (47). Notable members of this list include the AAA ATPase CDC48 and an E1 ubiquitin-activating enzyme (40, 41), which are homologs of the mammalian proteins p97 and UAE, respectively. Specific small-molecule inhibitors have been developed against both p97 and UAE (48–52), suggesting that their apicomplexan homologs might also be druggable. Of course, the apicoplast protein import pathway may house additional, yet-unexplored targets, including other plastid-localized components of the ERAD and ubiquitylation machinery, components of the TOC and TIC complexes, or proteins that have not yet been identified. Exploration of the mechanisms and druggability of apicoplast protein import may therefore be a useful strategy to identify drug targets.

## MATERIALS AND METHODS

### Ethics statement.

Human erythrocytes were purchased from the Stanford Blood Center (Stanford, CA) to support *in vitro* P. falciparum cultures. Because erythrocytes were collected from anonymized donors with no access to identifying information, IRB approval was not required. All consent to participate in research was collected by the Stanford Blood Center.

### Parasite growth.

P. falciparum Dd2^attB^ parasites (MRA-843) were obtained from MR4 and were grown in human erythrocytes (2% hematocrit) obtained from the Stanford Blood Center in RPMI 1640 medium (Gibco) supplemented with 0.25% AlbuMAX II (Gibco), 2 g/liter sodium bicarbonate, 0.1 mM hypoxanthine (Sigma), 25 mM HEPES (pH 7.4) (Sigma), and 50 μg/liter gentamicin (Gold Biotechnology) at 37°C, 5% O_2_, and 5% CO_2_.

### Vector construction.

Oligonucleotides (Table 1) were purchased from the Stanford Protein and Nucleic Acid facility. Molecular cloning was performed using In-Fusion cloning (Clontech). GFP and mDHFR were PCR amplified from pARL2-SBP1-mDHFR-GFP (26) using primers MB119/MB120 and MB121/MB122, respectively. These products were simultaneously cloned into the BsiWI/AflII sites of the plasmid pRL2-ACP_L_-GFP (53) or a similar plasmid containing the ACP_L_ K18E mutant (AAA-to-GAA codon change) to generate pRL2-ACP_L_-GFP-mDHFR and pRL2-ACP_L_(K18E)-GFP-mDHFR for expression of GFP-mDHFR fusions from the mitochondrial ribosomal protein L2 promoter (54).

**TABLE 1:** Primers used in this study

Name	Sequence	Description
MB119	TAAAAACCCACGTACGATGAGTAAAGGAGAAGAACTTTTC	Amplification of GFP to generate GFP-mDHFR fusion
MB120	TCGAACCATGGTACCTTTGTATAGTTCATCCATGCC	Amplification of GFP to generate GFP-mDHFR fusion
MB121	GGTACCATGGTTCGACCATTGAACTGC	Amplification of mDHFR to generate GFP-mDHFR fusion
MB122	ATAACTCGACCTTAAGTTAGTCTTTCTTCTCGTAGACTTCAAACTTATAC	Amplification of mDHFR to generate GFP-mDHFR fusion
TufA F	GATATTGATTCAGCTCCAGAAGAAA	Apicoplast genome qPCR; from Yeh and DeRisi (11)
TufA R	ATATCCATTTGTGTGGCTCCTATAA	Apicoplast genome qPCR; from Yeh and DeRisi (11)
CHT1 F	TGTTTCCTTCAACCCCTTTT	Nuclear genome qPCR; from Yeh and DeRisi (11)
CHT1 R	TAATCCAAACCCGTCTGCTC	Nuclear genome qPCR; from Yeh and DeRisi (11)

### Parasite transfection.

Transfections into Dd2^attB^ parasites were performed using a variation of the spontaneous uptake method (55, 56). Briefly, 50 μg each of pINT (30) and the desired pRL2 plasmid was ethanol precipitated and resuspended in an 0.2-cm electroporation cuvette in 100 μl TE buffer, 100 μl RPMI 1640 containing 10 mM HEPES-NaOH, pH 7.4, and 200 μl packed uninfected erythrocytes. Erythrocytes were pulsed with 8 square wave pulses of 365 V each of 1 ms separated by 0.1 s and were allowed to reseal for 1 h in a 37°C water bath before allowing parasites to invade. Drug selection with 2.5 μg/ml Blasticidin S (Research Products International) was initiated 4 days after transfection.

### Microscopy.

For live imaging, parasites were settled onto glass-bottomed microwell dishes (MatTek P35G-1.5-14-C) in PBS containing 0.4% glucose and 2 μg/ml Hoechst 33342 stain (ThermoFisher H3570).

For fixed imaging, parasites were processed as previously described (57) with modifications. Briefly, parasites were washed in PBS and fixed in 4% paraformaldehyde (Electron Microscopy Sciences 15710) and 0.0075% glutaraldehyde (Electron Microscopy Sciences 16019) in PBS for 20 min. Cells were washed once in PBS, resuspended in PBS, and allowed to settle onto poly-L-lysine-coated coverslips (Corning) for 1 h. Coverslips were washed once with PBS, permeabilized in 0.1% Triton X-100 in PBS for 10 min, and washed twice more in PBS. Coverslips were treated with 0.1 mg/ml sodium borohydride in PBS for 10 min, washed once in PBS, and blocked in 5% BSA in PBS. Following blocking, parasites were stained with 1:500 rabbit anti-*Pf*ACP (58) diluted in 5% BSA in PBS overnight at 4°C. Coverslips were washed three times in PBS, incubated for 1 h in 1:3,000 donkey anti-rabbit 568 secondary antibody (ThermoFisher A10042) in 5% BSA in PBS, washed three times in PBS, mounted onto slides with ProLong Gold antifade reagent with DAPI (ThermoFisher P36935), and sealed with nail polish prior to imaging.

Cells were imaged with a 100×, 1.35-NA objective on an Olympus IX70 microscope with a DeltaVision system (Applied Precision) controlled with SoftWorx version 4.1.0 and equipped with a CoolSnap-HQ CCD camera (Photometrics). Brightness and contrast were adjusted in Fiji (ImageJ) for display purposes.

To characterize apicoplast development following ACP_L_-GFP-mDHFR stabilization, parasites that were either untreated or treated with 10 nM WR99210 for 1 or 3 days were processed as above. ACP localization patterns from IFA images were classified into previously described categories (11, 33): “punctate,” “elongated,” “branched,” “divided,” or “diffuse.” At least 100 parasites were analyzed per condition per biological replicate. Parasites that were out of focus in images collected or that did not have detectable GFP expression were excluded from analysis.

### Parasite growth assays.

For dose-response assays, sorbitol-synchronized ring-stage parasites were grown in 96-well plates containing 2-fold serial dilutions of WR99210 (Jacobus Pharmaceutical Company) in the presence or absence of 200 μM IPP. After 3 days of growth, parasites were fixed in 1% paraformaldehyde in PBS and were stained with 50 nM YOYO-1 idide (ThermoFisher Y3601). Parasitemia was analyzed on a BD Accuri C6 flow cytometer. Each biological replicate of dose-response assays was performed in technical triplicate.

For time course growth experiments, sorbitol-synchronized parasites were untreated or were grown with 10 nM WR99210 with or without 200 μM IPP (Isoprenoids, LLC) for 5 days. Cultures were treated identically in terms of medium changes and splitting into fresh erythrocytes. Samples to assess growth were collected daily, fixed in 1% paraformaldehyde in PBS, and stored at 4°C until completion of the experiment. Samples were then stained with YOYO-1 and analyzed as above.

### qPCR.

Samples for DNA isolation were harvested daily during growth time course experiments. Parasites were released from erythrocytes by treatment with 0.1% saponin, washed in PBS, and stored at −80°C until analysis. Total parasite DNA was isolated using the DNeasy Blood & Tissue kit (Qiagen). qPCR was performed using Power SYBR Green PCR Master Mix (Thermo Fisher) with 0.15 µM each CHT1 F and CHT1 R primers targeting the nuclear gene chitinase or TufA F and TufA R primers targeting the apicoplast gene elongation factor Tu (11). qPCR was performed on Applied Biosystems 7900HT or ViiA 7 Real-Time PCR systems with the following thermocycling conditions: initial denaturation of 95°C for 10 min; 35 cycles of 95°C for 1 min, 56°C for 1 min, and 65°C for 1 min; and final extension of 65°C for 10 min. Relative quantification was performed using the ΔΔ*C_T_* method.

### Western blotting.

Sorbitol-synchronized, ring-stage parasites were either untreated, treated with 10 μM actinonin and 200 μM IPP for 3 days, or treated with 10 nM WR99210 and 200 μM IPP for 1 or 3 days. Parasites were separated from RBCs by lysis in 0.1% saponin, washed in PBS, and stored at −80°C until analysis. Parasite pellets were resuspended in PBS containing 1× NuPAGE LDS sample buffer with 50 mM DTT and were heated to 95°C for 10 min before separation on NuPAGE Bis-Tris gels and transfer to nitrocellulose. Membranes were blocked in 0.1% Hammarsten casein (Affymetrix) in 0.2× PBS with 0.01% sodium azide. Antibody incubations were performed in a 1:1 mixture of blocking buffer and TBST (Tris-buffered saline with Tween 20: 10 mM Tris, pH 8.0, 150 mM NaCl, 0.25 mM EDTA, 0.05% Tween 20). Blots were incubated with primary antibody at 4°C overnight at the following dilutions: 1:20,000 mouse anti-GFP JL-8 (Clontech 632381), 1:4,000 rabbit anti-*Pf*ClpP (59), and 1:20,000 rabbit anti-*P. falciparum* aldolase (Abcam ab207494). Blots were washed once in TBST and were incubated for 1 h at room temperature in 1:10,000 dilutions of IRDye 800CW donkey anti-rabbit or IRDye 680LT goat anti-mouse secondary antibodies (LI-COR Biosciences). Blots were washed three times in TBST and once in PBS before imaging on a LI-COR Odyssey imager. Band intensities of precursor and mature protein were quantified using Image Studio Lite version 5.2 (LI-COR).

### Statistics.

One-way ANOVAs with Tukey’s multiple-comparison tests were performed in GraphPad Prism version 7.04.
