# Supplementary figures and images for: Disruption of Apicoplast Biogenesis by Chemical Stabilization of an Imported Protein Evades the Delayed-Death Phenotype in Malaria Parasites
Source: mSphere. 2019 Jan 23;4(1):e00710-18. doi: 10.1128/mSphere.00710-18 (PMC6344605; doi:10.1128/mSphere.00710-18)

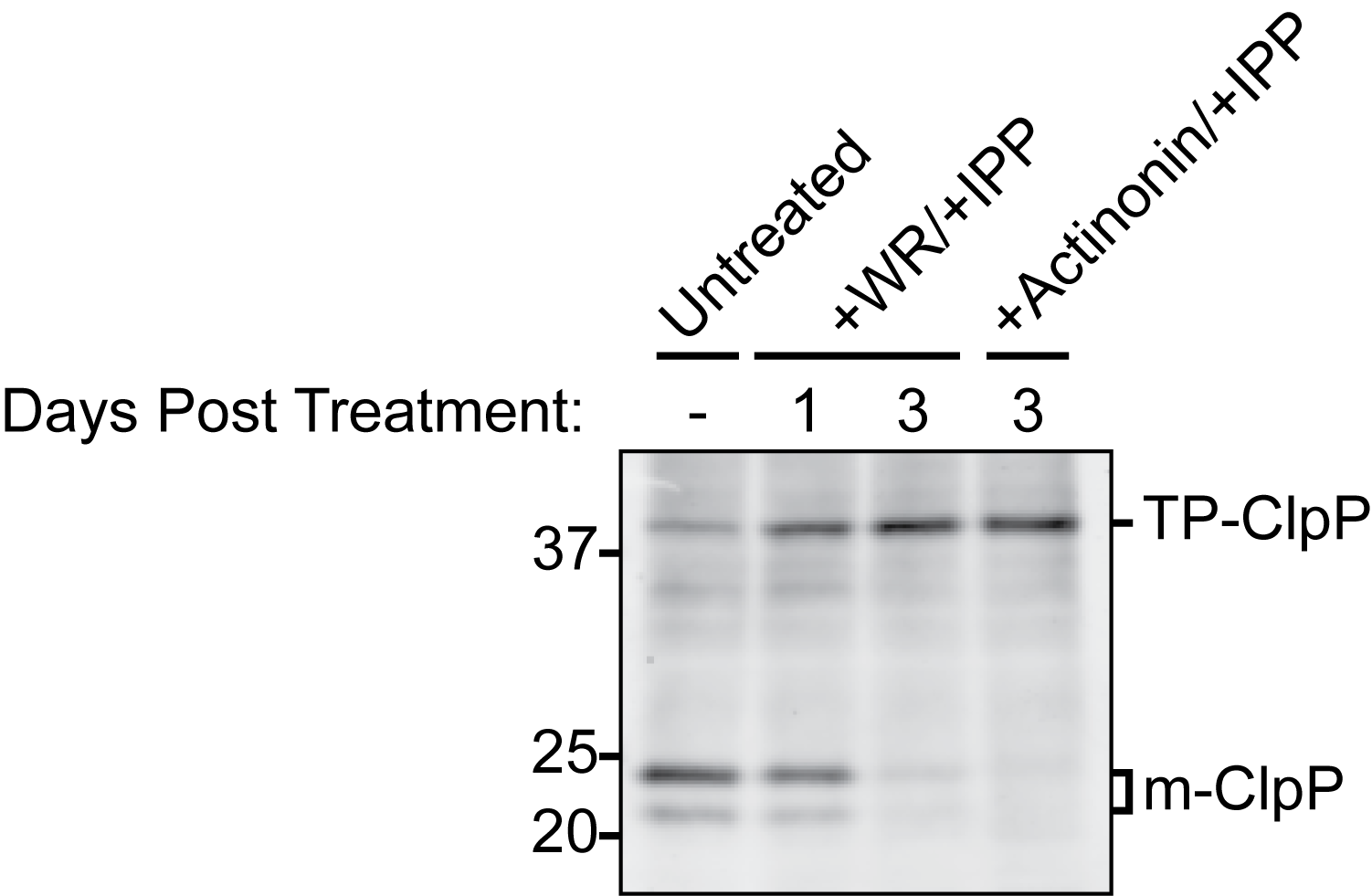

Supplement: FIG S1 [file mSphere.00710-18-sf001.tif]
